# Health-related quality of life in facial palsy: translation and validation of the Dutch version Facial Disability Index

**DOI:** 10.1186/s12955-020-01502-0

**Published:** 2020-07-31

**Authors:** Martinus M. van Veen, Tessa E. Bruins, Madina Artan, Tanja Mooibroek-Leeuwerke, Carien H. G. Beurskens, Paul M. N. Werker, Pieter U. Dijkstra

**Affiliations:** 1 Department of Plastic surgery, University of Groningen, University Medical Centre Groningen, P.O. Box 30.001, NL-9700 RB Groningen, the Netherlands; 2 Centre for Rehabilitation, University of Groningen, University Medical Centre Groningen, Groningen, the Netherlands; 3 Department of Orthopaedics, Physical Therapy section, Radboud University Medical Centre, Nijmegen, the Netherlands; 4 Department of Oral and Maxillofacial Surgery, University of Groningen, University Medical Centre Groningen, Groningen, the Netherlands

**Keywords:** Facial palsy, Facial disability index, Quality of life, Smallest detectable change

## Abstract

**Purpose:**

Patient-reported outcome measures are essential in the evaluation of facial palsy. Aim of this study was to translate and validate the Facial Disability Index (FDI) for use in the Netherlands.

**Methods:**

The FDI was translated into Dutch according to a forward-backward method. Construct validity was assessed by formulating 22 hypotheses regarding associations of FDI scores with the Facial Clinimetric Evaluation scale, the Synkinesis Assessment Questionnaire, the Short Form-12 and the Sunnybrook Facial Grading System. Validity was considered adequate if at least 75% (i.e. 17 out of 22) of the hypotheses were confirmed. Additionally, confirmatory factor analysis was performed. Cronbach’s α was calculated as a measure of internal consistency. Participants were asked to fill out the FDI a second time after 2 weeks to analyse test-retest reliability. Lastly, smallest detectable change was calculated.

**Results:**

In total, 19 hypotheses (86.4%) were confirmed. Confirmatory factor analysis showed acceptable fit for the two factor structure of the original FDI (root mean square error of approximation = 0.064, standardized root mean square residual = 0.081, comparative fit index = 0.925, Chi-square = 50.22 with 34 degrees of freedom). Internal consistency for the FDI physical function scale was good (α > 0.720). Internal consistency for the FDI social/well-being scale was slightly less (α > 0.574). Test-retest reliability for both scales was good (intraclass correlation coefficients > 0.786). Smallest detectable change at the level of the individual was 17.6 points for the physical function and 17.7 points for the social/well-being function, and at group level 1.9 points for both scales.

**Conclusion:**

The Dutch version FDI shows good psychometric properties. The relatively large values for individual smallest detectable change may limit clinical use. The translation and widespread use of the FDI in multiple languages can help to compare treatment results internationally.

## Introduction

Facial palsy results in functional and social problems related to the inability to control the muscles of facial expression [1–4]. Additionally, the altered facial function and appearance of the face may increase feelings of depression and anxiety, and may negatively influence self-image and quality of life [3–7]. The latter describes not so much the condition affecting the individual, but rather the individuals perception on their position in life including their social environment and mental health in the context of the condition. Evaluation of facial palsy should thus not only include facial movements and disabilities, but also include patient-perceived disability and quality of life.

The Facial Disability Index (FDI) is patient-reported outcome measure including ten-items, with a six-point ordinal answering scale [8]. Two FDI domain scores, the physical function and the social/well-being function, can be calculated ranging from 0 (worst) to 100 (best). Since the introduction of the original FDI in the 1990s, the FDI has been translated and validated to Spanish [9], Swedish [10], Italian [11], German [12], French [13], and Brazilian Portuguese [14]. However, previous studies did not include a pilot test stage [11, 12], pre-determined hypotheses for adequate construct validity [9, 10, 12–14], did not perform test-retest reliability [9, 12], and none determined smallest detectable change the FDI [9–14]. Aim of this study was to translate the FDI into Dutch and culturally validate the Dutch version of the FDI (FDI-NL) for use in Dutch speaking populations.

## Materials and methods

Our study protocol was reviewed by the medical ethics committee of our institution. The medical ethics committee deemed full and formal testing of our study protocol not necessary under current Dutch law. Patients from the outpatient departments of our institution provided written consent prior to participation. The developers of the original FDI granted permission to translate it into Dutch.

### Translation

**Fig. 1:**
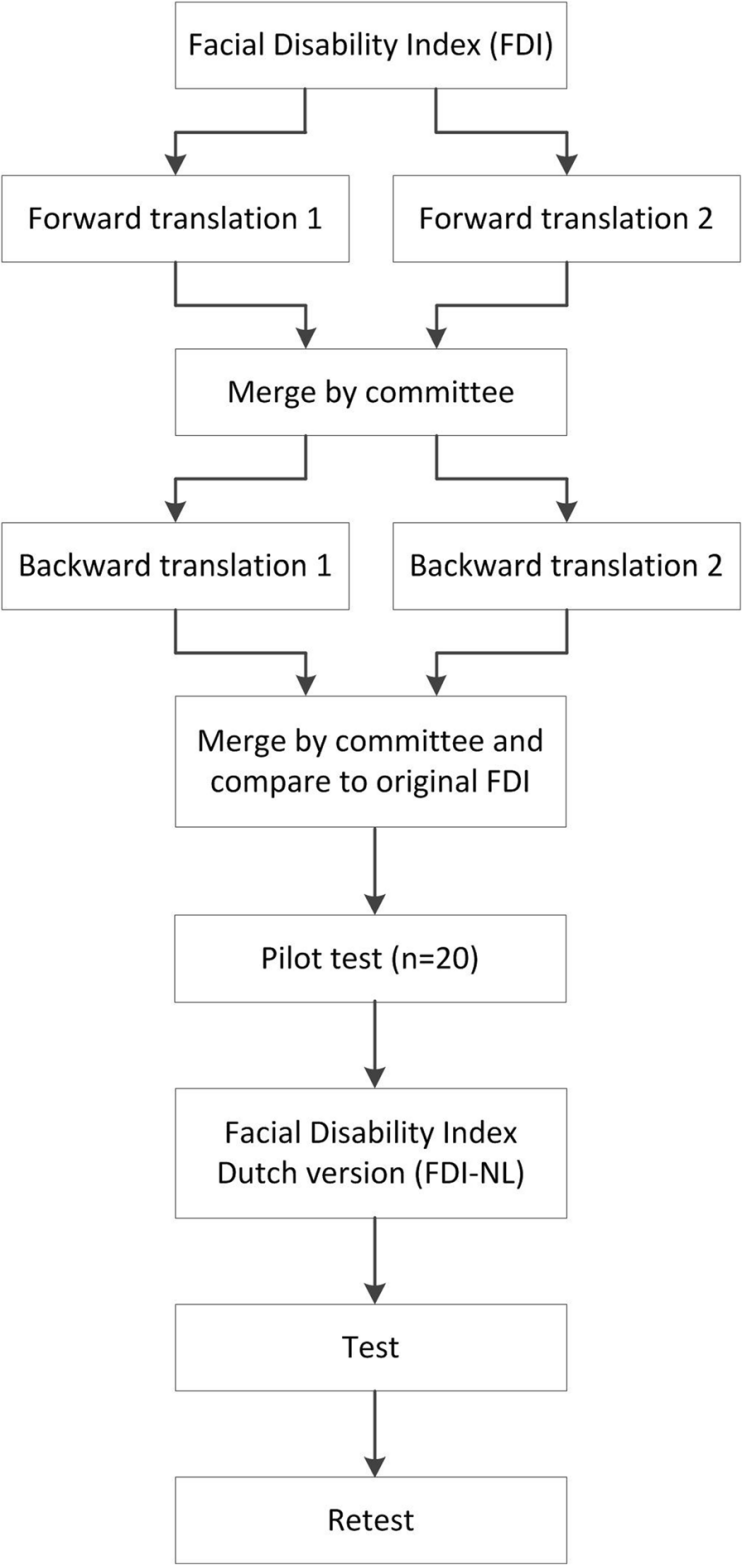
Schematic representation of the translation and validation process. Committee consisted of authors MMvV, PMNW and PUD. Pilot test was done with 10 facial palsy patients and 10 volunteers without facial disease. Retest moment was 2 weeks after test moment

The FDI-NL was created using a forward-backward translation method (Fig. 1). Two native Dutch speakers who are also fluent in English were asked to translate the English FDI into Dutch (B.t.H. and C.V., acknowledgements). A three-person committee (first, before last and last author, all native Dutch speakers with an excellent proficiency in English) with experience in the treatment of facial palsy and translating questionnaires then combined both forward translations into one consensus version FDI-NL. The consensus version was translated back to English by two native English speakers who were also fluent in Dutch (S.B. and N.T., acknowledgements). The same three-person committee compared the backward translations to the original FDI and the consensus version FDI-NL. A second consensus version FDI-NL was created and pilot tested in 10 patients with facial palsy and 10 healthy individuals. Pilot test participants were asked to critically review wording, phrasing and overall comprehensibility of the questionnaire, after which the final FDI-NL was constructed. Pilot testing was performed with 10 patients and 10 healthy individuals since facial palsy is relatively rare and the condition does not affect reading and language capabilities.

### Data collection

Adult patients with facial palsy who visited our department between January 2007 and January 2018 were invited to participate in our study. The patients were asked to visit our institution to fill out the questionnaires and measure current facial function. Patients fill out the questionnaires independently, without a researcher in the room.

### Construct validity

Validity of the FDI-NL was analysed by comparing FDI scores to several Dutch validated PROMs (Facial Clinimetric Evaluation scale (FaCE scale) [15, 16], Short Form 12 (SF-12) [17], the Synkinesis Assessment Questionnaire (SAQ)) [18, 19] and the Sunnybrook Facial Grading System (Sunnybrook) [20] as a measure of severity of facial palsy. The FaCE scale is a 15-item facial palsy-related quality of life questionnaire that comprises a total score and six domain score [15, 16]. The SF-12 is a measure of general health-related quality of life and comprises two domains: physical health and mental health [17]. The SAQ was used as a patient-reported measure of the severity of synkinesis [18, 19]. The Sunnybrook score was used to establish clinician-graded facial function [20]. Sunnybrook scoring was all done by one investigator (second author) based on a video from the clinic visit. Working hypotheses for the magnitude of the associations between the FDI-NL and FaCE, SF-12, SAQ and Sunnybrook scores were established based on those reported in the literature (Table 1) [9–13, 16, 19]. Based on the minimal and maximum reported associations we established a range in which we expected the associations to fall. We assumed adequate construct validity of the FDI-NL if 75% (i.e. 17 out of the 22) of hypotheses were confirmed [21].

**Table 1:** Expected range of associations between FDI-NL physical function and social/well-being function and FaCE scale, SF-12, SAQ and Sunnybrook scores

	*FDI-NL*	
	Physical function	Social/well-being function
**FaCE scale**		
Total score	0.50 < ρ < 0.80	0.45 < ρ < 0.70
Facial movement score	0.20 < ρ < 0.60	0.05 < ρ < 0.50
Facial comfort score	0.20 < ρ < 0.60	0.15 < ρ < 0.55
Oral function score	0.50 < ρ < 0.80	0.20 < ρ < 0.60
Eye comfort score	0.20 < ρ < 0.55	0.05 < ρ < 0.50
Lacrimal control score	0.15 < ρ < 0.45	0.05 < ρ < 0.40
Social function score	0.30 < ρ < 0.60	0.50 < ρ < 0.80
**SF-12**		
Physical health	0.20 < ρ < 0.55	0.15 < ρ < 0.55
Mental health	0.15 < ρ < 0.55	0.30 < ρ < 0.70
**SAQ**		
Total score	−0.50 < ρ < − 0.10	− 0.50 < ρ < − 0.10
**Sunnybrook**		
Total score	0.35 < ρ < 0.70	0.20 < ρ < 0.60

ρ: Spearman’s Rho

*Abbreviations*: *FaCE scale* Facial Clinimetric Evaluation scale, *FDI-NL* Dutch version Facial Disability Index, *SAQ* Synkinesis Assessment Questionnaire, *SF-12* Short Form 12, *Sunnybrook* Sunnybrook Facial Grading System

### Reliability

Reliability of the FDI-NL was examined by assessing internal consistency, item-total correlations and test-retest reliability for the FDI-NL scales. Internal consistency was examined at the test moment. Patients with a stable facial function (e.g. excluding patients in the recovery phase of Bell’s palsy or with reconstructive surgery planned in the near future) were asked to fill out the FDI-NL for a second time after 2 weeks to test for test-retest reliability of the FDI-NL.

The smallest detectable change (SDC) was calculated at an individual and group level to yield a value for FDI-NL scores after which change can be considered actual change, instead of measurement error. A SDC at the level of the individual was calculated (SDC_ind_) which can be used when interpreting change scores of one individual [21]. The group level SDC (SDC_group_) can be used to interpret changes at a group level [22].

### Statistical analysis

Statistical tests were performed in SPSS version 23 (IBM, New York, USA). Data is presented as frequencies and percentages, medians and interquartile ranges (IQR), and means and standard deviations (SD) as appropriate. Associations were analysed using Spearman’s rank correlation coefficients. A confirmatory factor analysis was performed using R software (version 3.4; R Foundation for Statistical Computing) to evaluate construct validity.

Cronbach’s α was calculated to analyse the internal consistency of the FDI-NL physical and social/well-being function scales. Additionally, Cronbach’s α was calculated for the FDI-NL scales with each item once excluded to evaluate if internal consistency would improve if that item was removed. Lastly, inter-item correlations were calculated to evaluate correlation between items.

Test-retest reliability was analysed using an intraclass correlation coefficient (ICC, two-way random effects model, single measures, absolute agreement). SDC was calculated in the following way. First the standard error of measurement (SEM_agreement_) was calculated by taking the square root of the error variance. Next, the SDC_ind_ was calculated using the formula: 1.96 x √2 x SEM_agreement_ [21]. The SDC_group_ was calculated by SDC_ind_ / √*n* [22]. Missing data for questionnaire items was estimated using multiple imputation.

## Results

### Questionnaire translation and pilot testing

The FDI-NL was created according to the above described steps. No problems in the wording and phrasing of the consensus version FDI-NL were identified during pilot testing. Seventeen out of 20 pilot test participants preferred to have the answer options presented in a long format instead of the two columns in the original version. For further testing the long format answer options were used (Appendix – FDI-NL final version).

### Study population

After pilot testing, 118 unilateral adult patients with facial palsy were included in this study. Eighty-seven (73.7%) patients also completed the retest FDI questionnaire 2 weeks after the visit to our institution. Sixty-two patients (52.5%) were female, median (IQR) age of the patients was 62.6 (48.8; 71.6) years. Most common cause of facial palsy was an acoustic neuroma (*n* = 29 (24.6%)), followed by trauma (*n* = 12 (10.2%)) (Table 2). All patients suffered from long-standing and irreversible facial palsy, and completed treatment for the underlying condition.

**Table 2:** Characteristics of study participants (*n* = 118)

Female gender (n (%))	62 (52.5)
Age, in years (median (IQR))	62.6 (48.8; 71.6)
Right sided facial palsy (n (%))	61 (51.7)
Duration of facial palsy, in years (median (IQR))	12.6 (6.6; 32.8)
Aetiology of facial palsy (n (%))	
Acoustic neuroma	29 (24.6)
Trauma	12 (10.2)
Congenital	11 (9.3)
Bell’s palsy	11 (9.3)
Parotid tumour	9 (7.6)
Iatrogenic	8 (6.8)
Brain tumour	8 (6.8)
Otitis media	6 (5.1)
Head and neck cancer	4 (3.4)
Unknown/other	20 (16.9)

*Abbreviations*: *IQR* interquartile range, *n* number

### Construct validity

Nineteen of the 22 validity associations (86.4%) were within the pre-determined range (Table 3). The correlations between both FDI-NL scales and the Sunnybrook total score and the FDI-NL physical function and FaCE Lacrimal Control subscale did not confirm our hypothesis.

**Table 3:** Correlations of FDI-NL scores with FaCE, SAQ, SF-12 and Sunnybrook scores

	FDI-NL Physical function (ρ (*p*-value))	Hypothesis confirmed?	FDI-NL Social/well-being function (ρ (*p*-value))	Hypothesis confirmed?
**FaCE scale**				
Total FaCE	0.661 (< 0.001)	Yes	0.511 (< 0.001)	Yes
Facial Movement	0.300 (0.001)	Yes	0.096 (0.315)	Yes
Facial Comfort	0.478 (< 0.001)	Yes	0.422 (< 0.001)	Yes
Oral Function	0.714 (< 0.001)	Yes	0.264 (0.004)	Yes
Eye Comfort	0.379 (< 0.001)	Yes	0.169 (0.069)	Yes
Lacrimal Control	0.090 (0.338)	No	0.103 (0.271)	Yes
Social Function	0.300 (0.001)	Yes	0.569 (< 0.001)	Yes
**SF-12**				
PCS	0.467 (< 0.001)	Yes	0.377 (< 0.001)	Yes
MCS	0.164 (0.085)	Yes	0.597 (< 0.001)	Yes
**SAQ**				
Total score	−0.243 (0.008)	Yes	−0.290 (0.001)	Yes
**Sunnybrook**				
Total score	0.072 (0.492)	No	0.023 (0.838)	No

ρ: Spearman’s Rho

*Abbreviations*: *FaCE scale* Facial Clinimetric Evaluation scale, *FDI-NL* Dutch version Facial Disability Index, *SAQ* Synkinesis Assessment Questionnaire, *SF-12* Short Form 12, *Sunnybrook* Sunnybrook Facial Grading System

Confirmatory factor analysis examining the fit of the original two latent factors of the FDI showed an acceptable level of fit for the Dutch version FDI with a root mean square error of approximation of 0.064, standardized root mean square residual of 0.081, comparative fit index of 0.925, and Chi-square value of 50.22 with 34 degrees of freedom [23–26]. Least fitting items were item 4 (‘How much difficulty did you have with your eye tearing excessively or becoming dry?’) in the physical function scale and item 8 (‘How much of the time did you get irritable toward those around you?’) in the social/well-being function scale (Table 4).

**Table 4:** Confirmatory factor analysis results

Observed variable	Latent variable	B	SE
FDI question 1	Physical	1.00	
FDI question 2	Physical	1.04	0.19
FDI question 3	Physical	0.75	0.16
FDI question 4	Physical	0.53	0.14
FDI question 5	Physical	0.90	0.17
FDI question 6	Social/Well-being	1.00	
FDI question 7	Social/Well-being	1.00	0.17
FDI question 8	Social/Well-being	0.28	0.11
FDI question 9	Social/Well-being	0.63	0.24
FDI question 10	Social/Well-being	1.06	0.19

*Abbreviations*: *B* coefficient, *SE* standard error

### Reliability

Internal consistency of the FDI-NL physical function scale was considered good, with a Cronbach’s α > 0.7. Cronbach’s α for the social/well-being function was 0.574 and 0.607 (Table 5). The ICC for test-retest reliability was good for both scales, with an ICC of 0.845 and 0.786 for the physical and social/well-being function respectively. On the 0 to 100 point FDI-NL scales, SDC_ind_ was 17.6 points for the physical function and 17.7 points for the social/well-being function. SDC_group_ was 1.9 points for both FDI scales (Table 6).

**Table 5:** Internal consistency measures overall Cronbach’s α, ‘Cronbach’s α if item deleted’ and inter-item correlations for all FDI-NL items on the test measurement moments

	Cronbach’s α		Inter-item correlation									
	Overall	If item deleted	Item 1	Item 2	Item 3	Item 4	Item 5	Item 6	Item 7	Item 8	Item 9	Item 10
***Physical function***	0.720											
Item 1		0.644	1.000	0.501	0.327	0.275	0.419	0.166	0.061	0.238	0.199	0.166
Item 2		0.638	–	1.000	0.409	0.180	0.471	0.095	0.060	0.207	0.273	0.184
Item 3		0.685	–	–	1.000	0.209	0.337	0.121	0.169	0.086	0.107	0.205
Item 4		0.736	–	–	–	1.000	0.272	0.241	0.061	0.059	0.316	0.153
Item 5		0.651	–	–	–	–	1.000	0.209	0.082	0.112	0.255	0.363
***Social/well-being function***	0.607											
Item 6		0.481	–	–	–	–	–	1.000	0.553	0.175	0.182	0.474
Item 7		0.464	–	–	–	–	–	–	1.000	0.232	0.196	0.489
Item 8		0.619	–	–	–	–	–	–	–	1.000	0.057	0.128
Item 9		0.686	–	–	–	–	–	–	–	–	1.000	0.174
Item 10		0.502	–	–	–	–	–	–	–	–	–	1.000

**Table 6:** Test-retest reliability and smallest detectable change

	Test-retest reliability		Smallest Detectable Change	
	ICC	95% CI	SDC_ind_	SDC_group_
Physical function	0.845	0.772; 0.897	17.6	1.9
Social/well-being function	0.786	0.688; 0.856	17.7	1.9

*Abbreviations*: *CI* confidence interval, *ICC* intraclass correlation coefficient, *SDC* smallest detectable change

Cronbach’s α was higher if item 4 (‘How much difficulty did you have with your eye tearing excessively or becoming dry?’) was deleted from the physical function scale, and if item 8 (‘How much of the time did you get irritable toward those around you?’) and item 9 (‘How often did you wake up early or wake up several times during your nighttime sleep?’) were deleted form the social/well-being function scale (Table 5). Inter-item correlations were deemed acceptable with the highest inter-item correlations within each subscale, and without highly correlated in general (Table 5).

## Discussion

The FDI–NL has good construct validity, test-retest reliability, and an acceptable internal consistency. Associations between the FDI-NL scales and Sunnybrook total scored below the expected range of correlations based on the literature. The association between FDI physical function and Sunnybrook was 0.63, 0.44 and 0.30 and 0.40, 0.19, and 0.21 between the FDI social/well-being function and Sunnybrook in the Swedish, Italian and French validation study respectively. We found a correlation of 0.072 and 0.023 respectively [10, 11, 13]. Hypothetically this is because we see relatively severe cases at our department, which might be different for the otolaryngology departments where the other studies were performed. The association between the FDI-NL and Sunnybrook was still positive, although much smaller than elsewhere reported. This difference may partly be due to the long duration of facial palsy time in our study. The median duration of facial palsy in our study was 12.4 years. Much longer compared to the 29 months in the validation study of the Dutch version FaCE scale [16], 22 months in the validation study of the Swedish version FDI [10], 140 days in the French validation study [13], and a mean duration of 3.5 years in the Italian version of the FDI validation study [11]. Patients with facial palsy may learn to cope with their disability over time and the association between patient-perceived disability and quality of life and a clinician-grading of facial palsy severity may change.

The internal consistency of the FDI-NL physical function scale was good with a Cronbach’s α above 0.7 at both the test and retest moment. The internal consistency of the social/well-being function scale was slightly less and did not reach the level of 0.7. Further analysis showed that removing item 9 (‘How often did you wake up early or wake up several times during your nighttime sleep?’) from the questionnaire would improve internal consistency of the scale the most. However, the median age of our study sample was 62.9 years compared to a mean age of 46.8 years in the original development study of the FDI [8]. The lower internal consistency caused by this question might be related to sleeping problems due to older age instead of a symptom of depression resulting from facial palsy. Additionally, removing item 4 from the questionnaire would increase the internal consistency of the physical function scale, although much less drastically. We believe this is related to the nature of the question itself; item 4 asks about eye-related complaints, while the other items are related to the mouth or midface. Perhaps further dividing the physical function scale into a scale related to the mouth and a scale related to the eye, such as in the FaCE scale [15], would have solved this issue. Removing item 8 from the questionnaire improved the internal consistency only slightly and only at the test moment. Since we did not develop the FDI, but solely translated and validated it for use in the Netherlands, we chose to keep the questionnaire as it is.

Similar to the internal consistency, we found items 4, 8 and 9 of the FDI-NL to be the least fitting items in our confirmatory factor analysis; most likely for the same reasons as described above. However, the FDI-NL as whole still showed an acceptable level of fit.

Test-retest reliability of the FDI-NL scales was good, with ICC point estimates of 0.845 and 0.786 and a confidence interval lower limit above 0.7 for the physical function scale. However, when using an instrument for individual decision making an ICC of 0.9 is advised [27]. We did not reach that level of test-retest reliability in our study. Recall bias because of the two-week time interval between the test and retest measurements could partly have influenced the ICC values. A two-week interval is generally considered as a margin to avoid recall bias, but short enough to avoid clinical improvement or deterioration [21].

The SDC is important for the interpretation of changes in scores. It indicates the point from which a change can be considered a true change and not due to measurement error. The FDI-NL SDC_group_ values were quite small in the present study. However, at an individual level the large SDC values of both the physical and social/well-being function limit clinical applicability. SDC values for other facial palsy-specific PROMs such as the FaCE scale and SAQ, are not reported yet and comparison is therefore impossible.

We did not perform a formal sample size calculation for this study. However, we assumed approximately 60 participants would be needed in our retest sample for adequate power of our test-retest reliability. Anticipating a participation rate of 50% in the retest, we set out to include approximately 120 patients in our study. Based on the literature, our actual sample size of 118 patients, with 87 retest participants, can be considered good to excellent [28, 29].

Although the FDI-NL knows several limitations, the developed Dutch version allows for objective measurement of patient-perceived disability and quality of life in a Dutch speaking population. Furthermore, it can be used to compare results to the international literature or to combine patient data from different countries. The larger values for the SDC_ind_ limit the use in clinical setting. Future research should focus on the development of a facial palsy-specific PROM that is well usable in individual follow up.

## Conclusion

The Dutch version FDI is a valid, reliable and easy to use questionnaire for the assessment of patient-perceived disability and quality of life in facial palsy. Although limited in clinical use in individuals, the FDI-NL provides the possibility to compare between clinics and so further increase knowledge about facial palsy and its effect on quality of life.

## Supplementary information

**Additional file 1.**

## Data Availability

The datasets used and/or analysed during the current study are available from the corresponding author on request.
