# Supplementary material for: Health-related quality of life in facial palsy: translation and validation of the Dutch version Facial Disability Index
Source: Health Qual Life Outcomes. 2020 Jul 31;18:256. doi: 10.1186/s12955-020-01502-0 (PMC7393719; doi:10.1186/s12955-020-01502-0)
Supplement: Supplementary file 1 — Additional file 1. [file 12955_2020_1502_MOESM1_ESM.docx]

**Facial Disability Index – Nederlandse versie (FDI-NL)**

Wilt u het meest geschikte antwoord kiezen op de volgende vragen die samenhangen met de functie van uw aangezichtsspieren? De vragen hebben betrekking op uw functioneren gedurende de afgelopen maand.

A.u.b. het gewenste antwoord omcirkelen.

Slechts **één** antwoord per vraag omcirkelen.

*Fysieke functie*

1. **Hoeveel problemen had u met voedsel in uw mond houden, voedsel in uw mond rond bewegen of voedsel dat blijft hangen in uw wang tijdens eten?**

*Meestal:*

5 geen problemen

4 weinig problemen

3 enige problemen

2 veel problemen

*Meestal niet gegeten vanwege:*

1 gezondheidsredenen

0 andere redenen

1. **Hoeveel moeite had u met het drinken uit een beker?**

*Meestal ging dit:*

5 zonder moeite

4 met een beetje moeite

3 met enige moeite

2 met veel moeite

*Meestal niet gedronken vanwege:*

1 gezondheidsredenen

0 andere redenen

1. **Hoeveel moeite had u met het uitspreken van bepaalde letters tijdens het praten?**

*Meestal ging dit:*

5 zonder moeite

4 met een beetje moeite

3 met enige moeite

2 met veel moeite, onduidelijke spraak

*Meestal niet gesproken vanwege:*

1 gezondheidsredenen

0 andere redenen

1. **Hoeveel last had u van een overmatig tranend oog of droog oog?**

*Meestal:*

5 geen last

4 weinig last

3 enige last

2 veel last

*Meestal geen tranen vanwege:*

1 gezondheidsredenen

0 andere redenen

1. **Hoeveel moeite had u met tandenpoetsen of mondspoelen?**

*Meestal ging dit:*

5 zonder moeite

4 met een beetje moeite

3 met enige moeite

2 met veel moeite

*Meestal geen moeite met poetsen of spoelen vanwege:*

1 gezondheidsredenen

0 andere redenen

*Sociale functie/welbevinden*

1. **Hoe vaak hebt u zich kalm en rustig gevoeld?**

6 altijd

5 meestal

4 vaak

3 soms

2 af en toe

1 nooit

1. **Hoe vaak hebt u zich geïsoleerd van mensen in uw omgeving?**

1 altijd

2 meestal

3 vaak

4 soms

5 af en toe

6 nooit

1. **Hoe vaak reageerde u geïrriteerd naar mensen in uw omgeving?**

1 altijd

2 meestal

3 vaak

4 soms

5 af en toe

6 nooit

1. **Hoe vaak werd u vroeg wakker of werd u ’s nachts verschillende keren wakker?**

1 elke nacht

2 de meeste nachten

3 een behoorlijk aantal nachten

4 sommige nachten

5 af en toe een nacht

6 nooit

1. **Hoe vaak heeft uw aangezichtsverlamming u ervan weerhouden uit eten te gaan, te winkelen of deel te nemen aan familie- of sociale activiteiten?**

1 altijd

2 meestal

3 vaak

4 soms

5 af en toe

6 nooit

| Opmerkingen: |
| --- |
